# Monitoring AI-generated English research texts: lexical-semantic accuracy, source fidelity, and revision performance

**DOI:** 10.3389/fpsyg.2026.1953783

**Published:** 2026-10-19

**Authors:** Jing Zhao, Lei Chen, Xi Bai

**Affiliations:** Department of Foreign Languages (International Department), Southwest Forestry University, Kunming, Yunnan, China

**Keywords:** AI-generated text, lexical-semantic accuracy, metalinguistic monitoring, revision performance, second language writing, source fidelity

## Abstract

**Introduction:**

Linguistically fluent AI-generated English texts can conceal lexical-semantic shifts, altered concept boundaries, source omissions, and unsupported causal formulations. This study compared English paragraphs generated under general and source-constrained conditions and examined how learners' global judgments and localized problem marking were associated with subsequent written revision.

**Methods:**

Seventy-three agricultural and forestry students evaluated two English paragraphs generated from Chinese source materials under a fixed sequence of general and source-constrained prompts, then selected and revised one paragraph. The linked corpus comprised 146 AI-generated texts, 73 revised texts, 146 global judgment records, 657 expert rating records, 562 coded terminology units, and 511 participant-marked problems. Expert assessments covered lexical-semantic accuracy, source fidelity, and global discourse organization, while revision analyses examined global judgment discrepancy, localized problem marking, and individual-difference variables.

**Results:**

Source-constrained texts received higher lexical-semantic accuracy scores (mean difference = 0.846, *t* = 10.634, *p* < 0.001, *d*_*z*_ = 1.245) and source-fidelity scores (mean difference = 0.855, *t* = 8.819, *p* < 0.001, *d*_*z*_ = 1.032), while global discourse organization differed little between conditions. Lexical-semantic accuracy was strongly associated with source fidelity (*b* = 0.522, *p* < 0.001), whereas the 5,000-resample bootstrap interval for the product of the adjusted coefficients linking these measures to discourse organization spanned zero. After controlling for selected source-text quality, localized problem marking and domain knowledge were positively associated with revised quality, trust in AI-generated language output was negatively associated, and global judgment discrepancy showed no unique association.

**Discussion:**

Source comparison and localized problem identification were more closely associated with successful written revision than agreement with expert global judgments. These findings highlight the importance of attending to linguistic form, semantic boundaries, and source information when evaluating and revising AI-generated English research texts.

## 1 Introduction

Large language models often produce English prose whose grammaticality, familiar vocabulary, and continuity create a strong impression of linguistic competence. Semantic reliability can vary beneath that fluent surface: generated passages may replace a technical expression with a broader label, shift a concept boundary, omit a source condition, or strengthen an associative statement into an unsupported causal claim (Bender et al., 2021; Ji et al., 2023). These changes matter when writers must evaluate a polished target-language text while retaining a precise representation of a source written in another language. Agricultural and forestry research offers a particularly informative context because organism names, indicators, experimental units, treatments, and result directions carry specific semantic commitments. AI-generated paragraphs derived from such materials expose a central language-psychological problem: how writers monitor plausible English when meaning must remain anchored to a constrained source.

This monitoring problem spans three levels of textual representation. Global discourse organization concerns paragraph coherence, information hierarchy, academic tone, and rhetorical progression. Lexical-semantic accuracy concerns whether English expressions preserve the identities and conceptual ranges of terms, variables, species, indicators, and units; it was assessed through expert terminology-accuracy ratings and term-level coding. Source fidelity concerns the preservation of evidence range, research object, section function, and causal commitment licensed by the Chinese source material, operationalized through expert disciplinary-fit ratings. L2 lexical research shows that form-to-meaning mappings may remain unstable even when wording appears familiar (Kroll and Stewart, 1994; Jiang, 2000, 2002), while discourse research separates local lexical knowledge from the construction of a coherent text-level representation (Perfetti and Stafura, 2014). A paragraph can therefore be well organized yet still misrepresent a term, variable, or evidential boundary.

Evaluating generated English after production can involve deliberate reflection on linguistic form, meaning, and appropriateness. Two observable behaviors are relevant to this monitoring process. Global judgment discrepancy is the absolute distance between a learner's overall quality score and the corresponding expert mean, whereas localized problem marking records overt annotations of terminological, semantic, evidential, methodological, or discourse problems. A learner may assign a plausible global score without locating language that requires revision; a marked problem provides a specific candidate for repair. Reflective, untimed language judgments have long been used to examine accessible explicit L2 knowledge (Ellis, 2004, 2005; Roehr, 2008; Plonsky et al., 2020). Combining global scoring with localized marking therefore permits a behavioral comparison between broad evaluative alignment and problem-specific attention.

Written revision carries monitoring into language production because the writer must compare representations, define a problem, select a repair, and formulate new English. Cognitive accounts place revision within a recursive coordination of planning, text generation, evaluation, and modification (Flower and Hayes, 1981; Fitzgerald, 1987). When writers work in English from Chinese source material, lexical knowledge, processing resources, and metacognitive control also shape how source meanings enter the revised text (Schoonen et al., 2003; Kormos, 2012). Each participant in the present task read a Chinese source package, judged two AI-generated English paragraphs, marked textual problems, selected one paragraph, and revised it in English. Expert-rated revised-text quality and change from the selected AI text provide complementary measures of the resulting written production.

Research on generative AI and English writing has documented variation in writing quality, feedback use, and learner engagement (Bauer et al., 2026; Pretorius and Thewissen, 2026; Yuan and Dao, 2026). Less is known about how source-sensitive properties of generated English relate to learners' subsequent judgments, problem localization, and revision. Linking AI texts, learner ratings, annotations, and revisions allows three connected questions to be examined. First, do source-constrained texts differ from general-prompt texts in lexical-semantic accuracy, source fidelity, and global discourse organization? Second, how closely are lexical-semantic accuracy and source fidelity related, and how does that relation compare with global organization? Third, do global judgment discrepancy and localized problem marking carry different information about subsequent written revision? This structure places learner language behavior and the semantic properties of generated text at the center of the analysis.

## 2 Related work

### 2.1 AI-generated text as linguistic input in second-language writing

Research on AI-mediated L2 writing encompasses the generation of initial text, automated feedback, machine assessment, and learner-led diagnosis and revision. These activities distribute linguistic judgment differently among the model, teacher, and learner. When an AI-generated paragraph becomes input for later comprehension and production, the learner must decide whether fluent English accurately represents the source. Reviews of natural language generation document how semantically unsupported content can coexist with locally plausible wording (Bender et al., 2021; Ji et al., 2023). Automation research identifies a related evaluative risk: imperfect automated guidance can attract omission and commission errors, while people may give algorithmic advice greater weight than advice from other people under some conditions (Parasuraman and Manzey, 2010; Logg et al., 2019). AI-literacy frameworks accordingly include knowledge of system capabilities and limitations and the critical evaluation of generated outputs (Long and Magerko, 2020; Ng et al., 2021). In source-based writing, source verification enacts those evaluative competencies through comparison of generated terms, claims, and evidential strength with the source before acceptance or revision (Keck, 2006; Wette, 2010).

Bauer et al. (2026) reported changes in writing quality, experience, and ownership when ChatGPT was embedded across an academic writing sequence. Focusing on a later production stage, Pretorius and Thewissen (2026) examined teacher-moderated ChatGPT feedback in relation to L2 revision accuracy and lexical diversity. By contrast, Zhou et al. (2026) evaluated reliability and feedback actionability when ChatGPT-4o scored second-language writing, placing the evaluative decision with the model. Together, these studies show how generated language enters drafting, feedback, and assessment, while leaving learner-led detection of source-related semantic deviation less fully explained.

The relevant sequence begins after an AI paragraph has been generated: the writer compares it with a Chinese source, judges its overall quality, marks localized problems, and produces an English revision. Recent studies have examined generative-AI-assisted EFL academic writing through an activity-theory perspective (Jia and Lu, 2026) and behavioral, cognitive, and emotional engagement during ChatGPT-assisted L2 revision (Yuan and Dao, 2026). Linking judgments, annotations, and revisions for the same participants and texts extends this work by testing whether global agreement with an expert score and problem localization provide distinct information about later written production.

### 2.2 Lexical-semantic accuracy and source-constrained discourse

Lexical-semantic accuracy concerns the mapping between an English expression and the concept it denotes in the source material. L2 lexical representations vary in the strength and route of their form–meaning connections, particularly when translation equivalents compete or a familiar English form activates a broader concept (Kroll and Stewart, 1994; Dijkstra and van Heuven, 2002). Jiang (2000) described L2 lexical development as a changing relation among lexical form, meaning, and conceptual representation. Extending this account, Jiang (2002) examined how form–meaning mappings develop during vocabulary acquisition. Applied to source-based writing, these models predict vulnerability when English terms must preserve specific variables, units, organisms, treatments, and conceptual boundaries.

Source fidelity operates across a broader propositional and rhetorical span because a research paragraph must retain what the source permits the writer to claim. Swales (1990) characterized research writing through genre-specific communicative moves, whereas Hyland (2008) emphasized disciplinary evidence, stance, and reader expectations. Source-based L2 writing research likewise distinguishes accurate paraphrase from changes that blur attribution or propositional content (Pecorari, 2003; Keck, 2006; Wette, 2010). Here, fidelity includes the preservation of research objects and result direction, restraint in causal wording, and compatibility with the target section. Changes to a treatment in Methods or the addition of an unsupported mechanism in Results and Discussion alter source meaning even when the paragraph remains coherent.

Lexical-semantic accuracy and source fidelity are related but distinct. Accurate terminology stabilizes reference to variables and concepts; fidelity additionally depends on omissions, unsupported additions, rhetorical function, and inferential scope. Global discourse organization forms a third level because information may be coherently ordered even when individual terms or claims depart from the source. The empirical question is whether lexical-semantic accuracy covaries with source fidelity while both remain distinguishable from paragraph organization.

### 2.3 Metalinguistic judgment and localized problem marking

Metalinguistic monitoring refers to reflective access to language as an object of judgment. Offline acceptability, interpretation, and rule-knowledge tasks are widely used to examine explicit L2 knowledge, with task format and response demands shaping the behavior observed (Ellis, 2004, 2005; Tokowicz and MacWhinney, 2005; Plonsky et al., 2020). Roehr (2008) linked metalinguistic knowledge with language ability among university-level L2 learners, identifying explicit language knowledge as a plausible resource for evaluating generated English. The global judgment discrepancy used here captures one observable outcome of that evaluation: the distance between a learner's overall score and an expert mean.

Localized problem marking provides a more specific record of monitoring. Levelt (1983) described monitoring and self-repair within speech production, while Postma (2000) compared accounts of how speakers detect production errors. Working with L2 speech, Kormos (1999) showed how a less automatized language system adds constraints to monitoring and self-repair. Across these accounts, global evaluation and the identification of a repairable segment perform different functions. An untimed written task transfers that distinction to overt post-generation judgments.

In an offline written task, learners can mark terminology problems, overgeneralized claims, unsupported additions, evidence-boundary violations, methodological details, and discourse-coherence problems. These annotations extend beyond form to semantic, evidential, disciplinary, and methodological comparisons with the source. Global judgment discrepancy and problem marking may share evaluative demands, yet the annotation identifies where revision could begin. L2 judgment research has established the relevance of explicit knowledge and task demands, but rarely tests whether global score alignment and localized source-based problem marking provide separable information about revisions of AI-generated text.

### 2.4 Written revision as language production

Revision is written language production in which a writer evaluates an existing text and transforms its linguistic form and propositional content. Flower and Hayes (1981) located reviewing within a recursive system of planning, translating, and monitoring, while Fitzgerald (1987) synthesized revision research across changes to meaning and surface form. Both accounts make problem representation central: the writer detects a discrepancy, determines its scope, and generates replacement language.

L2 revision adds demands from lexical access, syntactic formulation, and metalinguistic knowledge. Schoonen et al. (2003) compared first- and second-language writing through linguistic knowledge, processing speed, and metacognitive knowledge. Focusing on temporal allocation, Roca de Larios et al. (2008) examined how foreign-language writers distributed effort across writing processes, whereas Kormos (2012) reviewed individual differences in L2 written performance. Process-tracing research locates revisions through keystrokes, pauses, and text changes (Leijten and Van Waes, 2013). Completed judgments, annotations, and outcome scores offer a complementary view of learners' overt behavior after reading generated text.

AI-generated source texts create a distinctive production problem because learners begin with readable English whose wording may encourage retention. Engagement with corrective feedback varies across cognitive, behavioral, and affective dimensions (Han and Hyland, 2015). Recent work has examined teacher-moderated AI feedback during L2 revision (Pretorius and Thewissen, 2026) and behavioral, cognitive, and emotional engagement in ChatGPT-assisted revision (Yuan and Dao, 2026). Learner-led revision against a non-English source adds the need to compare lexical choices and claims with source information before reformulating the paragraph. The two prompt conditions provided different forms of source specification before global judgment and localized problem marking, which preceded a new act of English production.

## 3 Theoretical framework

### 3.1 AI-generated text as linguistically fluent but semantically uncertain input

Generated English may preserve readable paragraph structure while varying in the precision of its lexical choices and claims. The general prompt requested an academic paragraph from a Chinese source package, whereas the source-constrained prompt additionally specified the research object, terminology, variables, units, result direction, claim range, and section function. These additions target lexical-semantic accuracy and source fidelity directly; global discourse organization depends more strongly on how propositions are sequenced and connected. The three outcomes thus represent related levels of generated language with potentially different sensitivity to source information.

### 3.2 Lexical-semantic monitoring and source fidelity

Source information should matter most when meaning must be transferred from Chinese material to English output. Explicit terms and concept boundaries can stabilize reference to variables, units, organisms, and treatments, allowing the relevant entities and relations to remain identifiable across languages. Source fidelity also draws on omissions, additions, evidential scope, causal wording, and section function, so its relation to lexical-semantic accuracy should be close but not complete. Global discourse organization provides a parallel outcome: coherent sequencing may remain intact despite local semantic changes. This account predicts a closer lexical-semantic–fidelity relationship than either source-sensitive measure will show with paragraph organization.

### 3.3 Metalinguistic judgment, problem localization, and written revision

Revision begins with evaluation of a generated paragraph. Global judgment discrepancy reflects alignment between an overall score and expert judgment, while localized problem marking identifies expressions or claims that may require change. A marked problem provides a more explicit representation for revision because it connects evaluation with a location in the text. Revision then requires the learner to interpret the problem, compare the paragraph with the source, and formulate replacement language. Domain knowledge, writing self-efficacy, and trust in AI-generated language output may shape source comparison, confidence during production, and retention of generated wording.

The framework contains two behavioral sequences: source information is related to lexical-semantic accuracy and source fidelity, with global discourse organization treated as a parallel textual outcome; global judgment discrepancy and localized problem marking precede written revision. Solid arrows indicate the focal relations, and dashed arrows identify the parallel outcome and individual-difference covariates (Figure 1).

**Figure 1:**
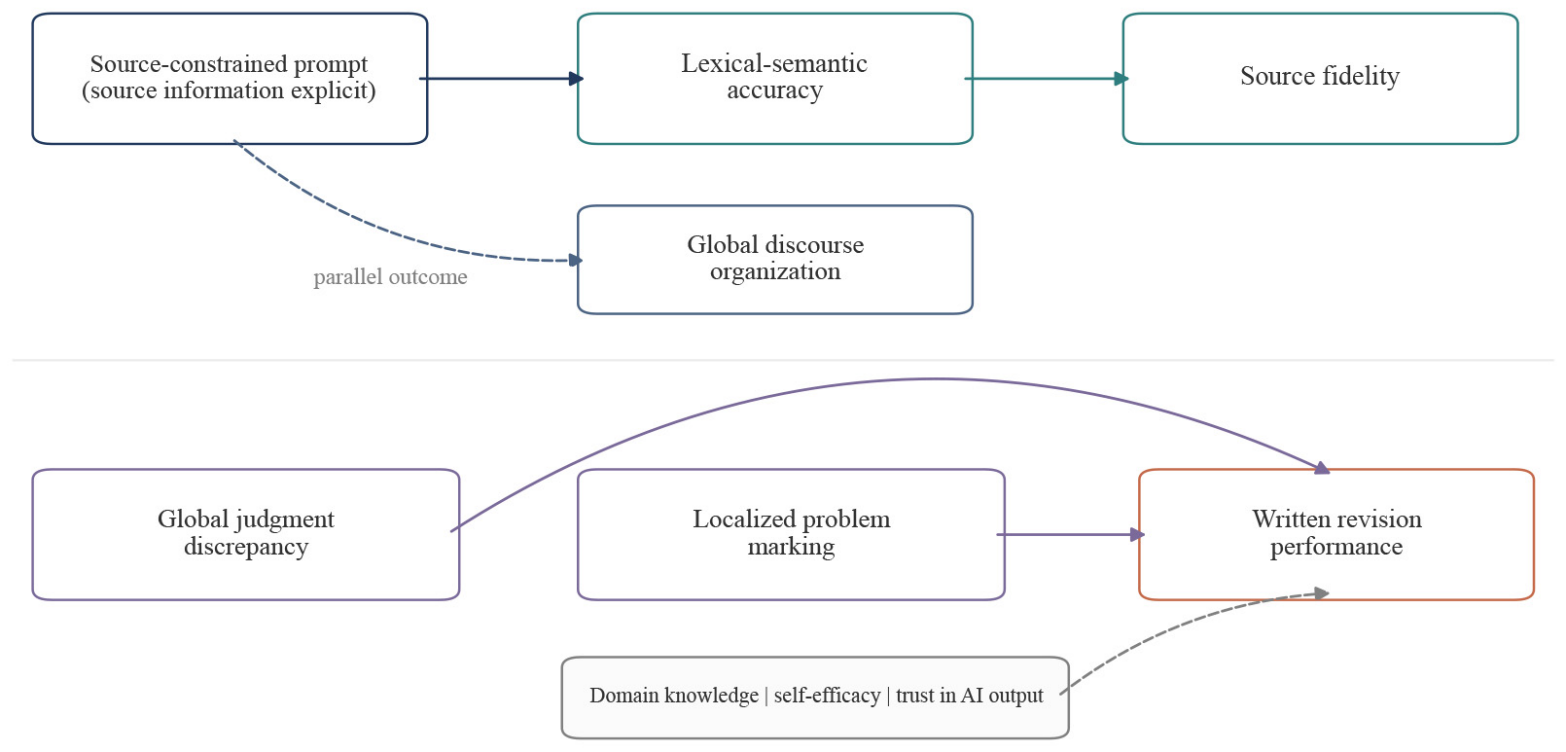
Behavioral framework for monitoring and revision of AI-generated English research text. The upper sequence relates source information to lexical-semantic accuracy and source fidelity, with global discourse organization assessed in parallel. The lower sequence relates global judgment discrepancy and localized problem marking to subsequent written revision. Solid arrows denote focal associations; dashed arrows denote the parallel outcome and individual-difference covariates.

These relations yield three research questions:

**RQ1:** do texts produced in the source-constrained condition differ from texts produced in the general condition in lexical-semantic accuracy, source fidelity, and global discourse organization?
**RQ2:** to what extent is lexical-semantic accuracy associated with source fidelity, and is this relationship distinguishable from global discourse organization?
**RQ3:** how are global judgment discrepancy and localized problem marking associated with subsequent written revision performance?

The conceptual definitions and operational measures of the six research constructs are summarized in Table 1.

**Table 1:** Language-related constructs and operational measures.

Construct	Conceptual definition	Operational indicator
Global discourse organization	Paragraph-level coherence, information hierarchy, academic tone, and rhetorical progression in written production (Flower and Hayes, 1981; Swales, 1990).	Expert discourse-quality rating on a seven-point scale, averaged across three raters
Lexical-semantic accuracy	Preservation of term meaning, variable identity, units, species reference, and concept boundaries across languages (Kroll and Stewart, 1994; Jiang, 2000).	Expert terminology-accuracy rating, supplemented by term-level error coding
Source fidelity	Preservation of source evidence, research object, section function, and warranted causal scope (Hyland, 2008; Keck, 2006; Wette, 2010).	Expert disciplinary-fit rating on a seven-point scale, averaged across three raters
Global judgment discrepancy	Distance between a learner's offline global quality judgment and expert judgment (Flavell, 1979; Ellis, 2004).	Absolute student–expert score discrepancy; signed bias is reported separately
Localized problem marking	Overt marking of a localized lexical, semantic, evidential, methodological, or discourse problem (Levelt, 1983; Kormos, 1999).	Count and recorded type of participant annotations
Written revision performance	Expert-rated quality of a new English paragraph and its change from the selected AI source text (Fitzgerald, 1987; Schoonen et al., 2003).	Raw revised-text overall-quality score as the primary outcome; revised minus selected-source score as a secondary gain index

## 4 Methods

### 4.1 Participants and task setting

Seventy-three participants enrolled in agricultural and forestry-related programs completed the behavioral judgment-and-revision task in a classroom setting. Their majors were Plant Protection, Agricultural Resources and Environment, Agronomy, Horticulture, Forestry, Ecology, Food Science, and Smart Agriculture. Participants worked from Chinese disciplinary source materials to evaluate and revise English research paragraphs. Anonymized IDs linked each source package with the generated texts, global judgments, problem annotations, revised text, and expert ratings. Participation did not determine course grades.

Participant characteristics available for analysis included major, study level, assigned writing section, AI-use frequency, domain knowledge, writing self-efficacy, and trust in AI-generated language output. Table 2 shows representation across third-year undergraduate, fourth-year undergraduate, and first-year master's study, with similar numbers assigned to Introduction, Methods, and Results and Discussion paragraphs. This distribution supported writing section as a task covariate and domain knowledge as an individual-difference covariate in the revision models.

**Table 2:** Participant characteristics and writing task allocation.

Characteristic	Category or statistic	Value
Major	Plant Protection	14
	Agricultural Resources and Environment	13
	Agronomy	12
	Horticulture	12
	Forestry	8
	Ecology	6
	Food Science	5
	Smart Agriculture	3
Grade	Year 4 undergraduate Master year 1	29 23
	Year 3 undergraduate	21
Writing task section	Methods	25
	Introduction	24
	Results and Discussion	24
AI use frequency	Mean (SD)	2.68 (0.89)
Domain knowledge score	Mean (SD)	70.05 (10.50)
Writing self-efficacy	Mean (SD)	3.22 (0.62)
Trust in AI-generated language output	Mean (SD)	3.23 (0.63)

Full item wording and scoring rules for the recorded individual-difference measures are provided in Supplementary Tables S1–S8.

### 4.2 AI text generation and behavioral procedure

Each participant used the Doubao web interface to generate two English research paragraphs from one Chinese source-material package. The general prompt requested an academic English paragraph. The source-constrained prompt additionally required preservation of experimental objects, variables, treatment conditions, units, result direction, field-specific terms, source-supported claim range, and the rhetorical function of the assigned section. All participants completed the general-prompt condition first and the source-constrained condition second; condition contrasts therefore refer to this fixed within-participant sequence.

The general prompt requested a 170–210-word paragraph for the assigned article section (Introduction, Methods, or Results and Discussion), using formal academic English and returning only the paragraph. The source-constrained prompt used the same length, section, language, and output-format requirements and added explicit constraints on research objects, terminology, variables, treatments, values, units, result direction, evidence range, causal wording, and section function. It also supplied a corresponding terminology list. Full prompt templates and generation-control information are provided in Supplementary Tables S20–S21.

Doubao was accessed through its standard web-based text-dialogue interface during 23–27 March 2026 (Beijing time, UTC+8). Each formal generation began in an independent conversation with zero prior turns, and the resulting output was evaluated without manual editing. The interface did not expose a stable backend model ID or sampling controls; the 170–210-word instruction supplied the output-length constraint. Six technical retries occurred among 146 generation requests (four network interruptions and two truncated outputs); each retry resubmitted the identical prompt, and the first complete output was used for evaluation. Prompt, output, condition, material, participant, timestamp, and retry information were recorded with the generation materials. The full generation record and platform-specific details are provided in Supplementary Tables S20–S21.

After generating both English texts, participants assigned an overall-quality score to each paragraph and marked localized language problems. They then selected one text and revised it in English. This procedure yielded two generated texts and two judgment records per participant, followed by one revision record. Fifty-eight participants selected a source-constrained text and 15 selected a general-prompt text. The primary revision model used revised-text quality as the outcome and selected source-text quality as a covariate; revision gain supplied an alternative parameterization of the same adjusted relation. Both specifications included selected prompt condition, initial terminology-error count, writing section, and material package where applicable.

Figure 2 summarizes the resulting data structure. Each participant was linked to one source package and writing-section function, two prompt-condition texts, two global judgments, problem annotations, one selected source text, and one revised text. Revision-model predictors were drawn from the selected text: judgment discrepancy and annotation count refer to the paragraph each participant revised. Material package and writing section entered the models as observed design covariates. This nesting separates the paired text-level condition comparison from the participant-level revision analysis.

**Figure 2:**
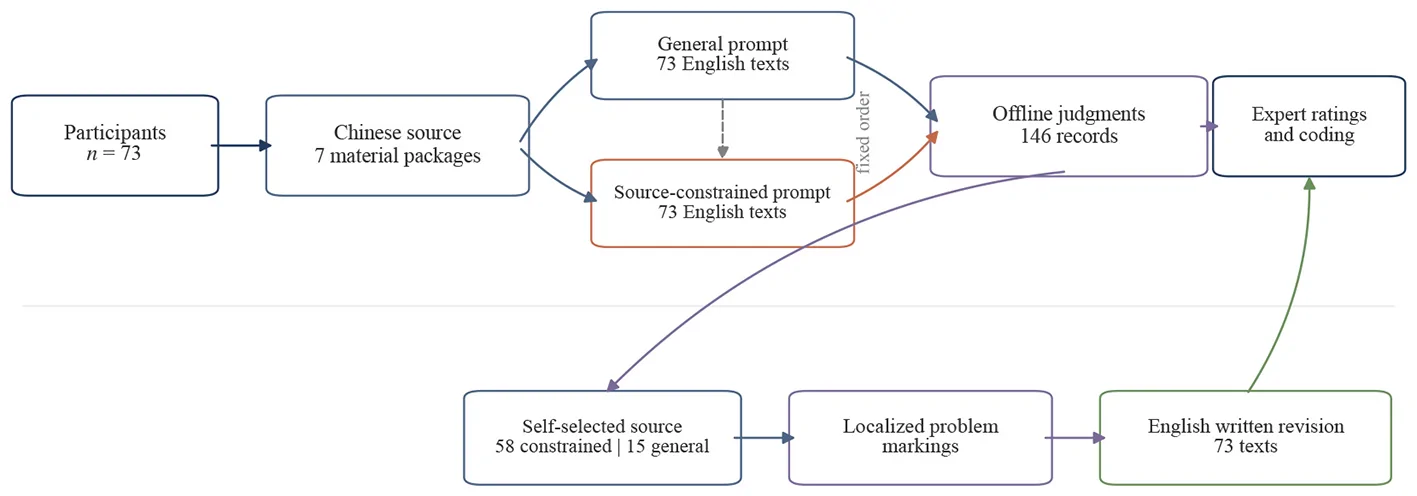
Behavioral judgment-and-revision procedure and nesting of analysis records. Each participant generated one English text from a Chinese source package under the general prompt and then one under the source-constrained prompt, judged both texts, marked localized language problems, and revised one self-selected text. The paired condition analysis contains two texts per participant; the revision analysis contains one selected source and one revised text per participant.

### 4.3 Source materials and corpus construction

Seven Chinese source packages represented paragraph-level research texts in agriculture and forestry: salt stress and antioxidant enzyme activity in maize seedlings; nitrogen-gradient effects on wheat yield and nitrogen-use efficiency; biochar amendment of acidic soil and rice root growth; understory light and photosynthetic traits of forest seedlings; fungal infection and leaf defense responses in crop plants; drought responses of fruit-tree rootstocks; and urban plant diversity and soil microbial communities. Each package specified a target article section, research objects, variables, treatment or contextual information, result direction, and a terminology list. These constraints made changes in English terms, reference, evidence range, and causal wording identifiable against the source.

The corpus comprised 73 participant cases, 146 generated English paragraphs, and 73 revised paragraphs. Three experts produced 438 ratings of generated texts and 219 ratings of revisions, while two coders produced 1,124 records from 562 term units. The 511 problem annotations were nested within participants and generated texts. Sentence-level units and reflection records were also recorded; the linked text, revision, judgment, terminology, and annotation records formed the basis of the inferential analyses (Table 3).

**Table 3:** Corpus composition and coding units.

Data component	Unit description	Count
Participants	Participant records	73
AI-generated texts	Two prompt conditions per participant	146
Participant-revised texts	One revision per participant	73
Global judgment records	One record per AI text	146
AI expert rating records	146 texts × 3 raters	438
Revision expert rating records	73 revisions × 3 raters	219
Terminology consensus units	Term-level units	562
Terminology coder records	Two coders per term unit	1,124
Localized problem annotations	Participant-marked issues	511
Sentence-level coding units	AI and revised texts	1,859
Reflection records	One reflection per participant	73

### 4.4 Measures and coding scheme

Three experts rated each generated English text on seven-point scales for discourse quality, terminology accuracy, disciplinary fit, and overall quality. Global discourse organization was assessed from paragraph structure, coherence, information hierarchy, and academic tone. Lexical-semantic accuracy was assessed from terminology accuracy, with term-level coding providing additional information about lexical and conceptual deviations. Source fidelity was operationalized through the disciplinary-fit rubric, which emphasized preservation of source evidence, research objects, section function, and warranted claims. The separate dimensions allowed a coherent paragraph to receive a lower score when its terminology or claims departed from the source. AI-generated texts and participant revisions were rated in separate sets or stages. Raters knew whether a text was an AI-generated paragraph or a participant revision, were blind to the general versus source-constrained condition, and received texts in randomized order within each set.

The complete participant and expert scoring anchors are provided in Supplementary Tables S9–S13.

Writing self-efficacy was calculated as the mean of four recorded items (WSE1–WSE4). Trust in AI-generated language output was calculated as the mean of four recorded items (TR1–TR4); TR3 was reverse scored during data preparation. Item wording, response anchors, and scoring rules are provided in Supplementary Tables S1–S5.

Revised texts were rated on the same three language dimensions and overall written quality. Raw revised-text overall quality served as the primary outcome in the baseline-adjusted model, while Equation 2 supplied an alternative change-score parameterization. Two coders separately identified terminology errors, including mistranslation, vague generalization, collocation problems, unit or indicator errors, species-expression errors, concept drift, and unsupported information. Participant markings followed seven recorded categories: terminology issue, disciplinary-fit issue, overgeneralized claim, discourse-coherence issue, method/detail issue, unsupported information, and evidence-boundary issue. The sum of these markings formed the localized problem count.

The terminology and participant-marking codebooks, counting rules, and ambiguity examples are provided in Supplementary Tables S14–S17.

Global judgment discrepancy was calculated as the absolute distance between each participant's overall quality rating and the corresponding expert mean, as defined in Equation 1:


$$\begin{array}{l} \text{JudgmentDiscrepanc}{\text{y}}_{ij}=|\text{StudentScor}{\text{e}}_{ij}-\text{ExpertScor}{\text{e}}_{ij}|, \end{array}$$ (1)

In Equation 1, *i* indexes the participant and *j* the AI-generated text evaluated by that participant. Larger values indicate greater distance from expert judgment. Signed judgment bias was also calculated as StudentScore_*ij*_−ExpertScore_*ij*_ to retain direction: positive values indicate overestimation and negative values underestimation. The revision model used the discrepancy value for the text each participant selected to revise.

Written revision performance gain was calculated as the difference between the expert-rated overall quality of the revised paragraph and that of the selected source text, following Equation 2:


$$\begin{array}{r} \text{RevisionPerformanceGai}{\text{n}}_{i}=\text{ExpertScore}\left(\text{RevisedTex}{\text{t}}_{i}\right)\\ -\text{ ExpertScore}\left(\text{AITex}{\text{t}}_{i}\right). \end{array}$$ (2)

In Equation 2, positive values indicate higher expert-rated quality after revision, zero indicates no score change, and negative values indicate lower quality after revision. The index *i* refers to the participant-level record. The calculation uses the AI text that the participant selected and revised; it is distinct from the paired difference between general and source-constrained generation conditions. Because revision gain equals revised-text quality minus selected source-text quality, the gain specification provides the change-score parameterization of the primary baseline-adjusted model: coefficients for the other predictors are unchanged, while the source-quality coefficient is shifted by −1.

Table 4 summarizes the scoring structure across the three language levels. Separate ratings for global organization, lexical-semantic accuracy, and source fidelity distinguished paragraph coherence from meaning preservation. Revision descriptors covered effective correction and information fidelity; the inferential models use overall written quality and its derived gain index.

**Table 4:** Rating rubric for global discourse organization, lexical-semantic accuracy, source fidelity, and written revision quality.

Dimension	Subdimension	Scoring description	Scale
Global discourse organization	Paragraph organization	Clarity of topic, development, and closure	1–7
	Logical coherence	Consistency of sentence-to-sentence reasoning	1–7
	Information hierarchy	Appropriate prioritization of background, method, results, and interpretation	1–7
Lexical-semantic accuracy	Term translation	Accuracy of source-to-English term mapping	1–7
	Indicator and unit accuracy	Correct expression of measures, units, and variables	1–7
	Concept boundary	Avoidance of concept drift and vague generalization	1–7
Source fidelity	Agricultural/forestry context	Preservation of field-specific source meaning and section function	1–7
	Evidence boundary	Avoidance of unsupported causal or mechanistic claims	1–7
Written revision quality	Effective correction	Improvement in lexical-semantic accuracy, discourse organization, and source fidelity	1–7
	Information fidelity	Retention of source content without adding unsupported information	1–7

All recorded dimensions use a seven-point scale.

Coder training and formal coding procedures are provided in Supplementary Tables S18–S19.

### 4.5 Data analysis

Descriptive statistics were computed for the three language outcomes, word count, terminology error count, global judgment discrepancy, signed judgment bias, and localized problem count by condition. Judgment discrepancy from Equation 1 and selected-text revision gain from Equation 2 were linked to the participant-level revision record. Internal consistency for writing self-efficacy and trust in AI-generated language output was estimated with Cronbach's α and McDonald's ω (Cronbach, 1951; McDonald, 1999). Expert inter-rater agreement was estimated with ICC(2,k) for the mean of three ratings (Shrout and Fleiss, 1979), and terminology-code agreement with Krippendorff's α (Hayes and Krippendorff, 2007). Paired *t* tests, Wilcoxon signed-rank tests, 95% confidence intervals, and Cohen's *d*_*z*_ quantified within-participant condition differences; bootstrap intervals for *d*_*z*_ used 5,000 participant-level resamples. Variables used in the reported models had complete records. All tests were two-sided with α = 0.05, and reported *p* values were unadjusted. Confidence intervals, effect sizes, and distributional patterns were considered alongside statistical significance.

To estimate the condition difference while accounting for writing section, material-package differences, and paired observations, the cluster-robust model in Equation 3 was fitted separately for global discourse organization, lexical-semantic accuracy, and source fidelity:


$$\begin{array}{l} Y_{ij}=\beta_{0}+\beta_{1}\text{SourceConstrain}{\text{t}}_{ij}+\beta_{2}\text{Sectio}{\text{n}}_{i}\\ \;+\;\beta_{3}\left(\text{SourceConstrain}{\text{t}}_{ij}\times \text{Sectio}{\text{n}}_{i}\right)+\gamma_{m}\text{Materia}{\text{l}}_{m}+\varepsilon_{ij}. \end{array}$$ (3)

In Equation 3, *Y*_*ij*_ is one expert-rated outcome for participant *i* and AI text *j*. SourceConstraint_*ij*_ was coded 1 for the source-constrained prompt and 0 for the general prompt. Section_*i*_ represented Introduction, Methods, or Results and Discussion, with Introduction as the reference category. The interaction tested whether the condition difference varied by section, γ_*m*_ represented material-package fixed effects, and ε_*ij*_ was the residual. Standard errors were clustered by participant because each person contributed two texts. With the interaction present, β_1_ represents the condition difference in the Introduction reference section; section-specific effects and an additive model were reported separately.

Text-level regressions predicted source fidelity from lexical-semantic accuracy and global discourse organization from lexical-semantic accuracy and source fidelity, controlling for word count, writing section, and material package. All text-level standard errors were clustered by participant. The product of the two adjusted coefficients was estimated with 5,000 participant-level bootstrap draws. The primary participant-level model regressed raw revised-text overall quality on selected-text global judgment discrepancy from Equation 1, selected-text localized problem count, writing self-efficacy, trust in AI-generated language output, domain knowledge, selected source quality, selected prompt condition, initial terminology-error count, writing section, and material package. The gain specification provided the change-score parameterization of the same adjusted relation. Model fit, parameter counts, HC3 robust intervals, and variance-inflation factors were reported for the 17-parameter specification. Functional form, heteroskedasticity, residual magnitude, and influential observations were examined with Ramsey RESET, joint quadratic-term, Breusch–Pagan, studentized-residual, Cook's *D*, and leverage diagnostics. The complete coefficient and diagnostic summaries are provided in Supplementary Tables S22–S23. Analyses and figures were produced in Python with pandas, NumPy, SciPy, statsmodels, Matplotlib, and seaborn (Harris et al., 2020; Virtanen et al., 2020).

## 5 Results

### 5.1 Descriptive statistics and reliability

The condition difference was concentrated in meaning preservation rather than paragraph organization (Table 5). Global discourse organization scores were similar under the general prompt (M = 4.48, SD = 0.42) and source-constrained prompt (*M* = 4.61, SD = 0.45). Lexical-semantic accuracy, assessed through terminology-accuracy ratings, was higher in the source-constrained condition (*M* = 4.73, SD = 0.50) than in the general condition (*M* = 3.89, SD = 0.65). Source fidelity, assessed through disciplinary-fit ratings, showed a comparable separation (*M* = 4.80, SD = 0.60 vs. *M* = 3.94, SD = 0.52). Mean terminology error counts were 1.10 and 2.42, respectively. Source-constrained texts were also slightly longer, but the largest descriptive differences concerned lexical-semantic and source-based properties.

**Table 5:** Descriptive statistics and consistency coefficients.

Measure	General prompt	Source-constrained prompt
Panel A. Descriptive statistics by prompt condition		
Global discourse organization	4.48 (0.42)	4.61 (0.45)
Lexical-semantic accuracy	3.89 (0.65)	4.73 (0.50)
Source fidelity	3.94 (0.52)	4.80 (0.60)
Overall quality	4.10 (0.35)	4.71 (0.33)
Word count	182.07 (24.05)	192.15 (24.10)
Terminology error count	2.42 (1.71)	1.10 (1.26)
Global judgment discrepancy	0.33 (0.29)	0.24 (0.20)
Localized problem count	3.01 (1.87)	2.19 (1.70)
Panel B. Reliability coefficients and inter-rater agreement		
Scale or rating	Statistic	Value
Writing self-efficacy (four items)	Cronbach's α	0.826
Writing self-efficacy (four items)	McDonald's ω	0.886
Trust in AI-generated language output (four items)	Cronbach's α	0.808
Trust in AI-generated language output (four items)	McDonald's ω	0.876
Global discourse organization	ICC(2,k)	0.863
Lexical-semantic accuracy	ICC(2,k)	0.924
Source fidelity	ICC(2,k)	0.926
Revision overall quality	ICC(2,k)	0.922
Terminology error type	Krippendorff's α	0.803

Panel A reports means (SDs) by generation condition. Panel B reports scale internal consistency, expert inter-rater agreement, and terminology-coding agreement. ICC, intraclass correlation coefficient; α, Cronbach's or Krippendorff's alpha as indicated; ω, McDonald's omega.

Expert agreement was high for global discourse organization (ICC(2,k) = 0.863), lexical-semantic accuracy (ICC(2,k) = 0.924), source fidelity (ICC(2,k) = 0.926), and revision overall quality (ICC(2,k) = 0.922). Terminology coding reached Krippendorff's α = 0.803. Internal consistency was satisfactory for the four-item writing self-efficacy scale (Cronbach's α = 0.826, McDonald's ω = 0.886) and the four-item trust in AI-generated language output scale (Cronbach's α = 0.808, McDonald's ω = 0.876) (Table 5).

The distributions reinforce this asymmetry: global discourse organization showed substantial overlap between conditions, including similar central ranges and many participant pairs with modest differences, whereas lexical-semantic accuracy and source fidelity displayed clearer upward shifts in their medians and fewer low-scoring texts in the source-constrained condition (Figure 3). Faint within-participant links make the paired structure visible while individual observations remain dispersed within both conditions. The strongest separation therefore occurred in meaning preservation, although it was not uniform across every text.

**Figure 3:**
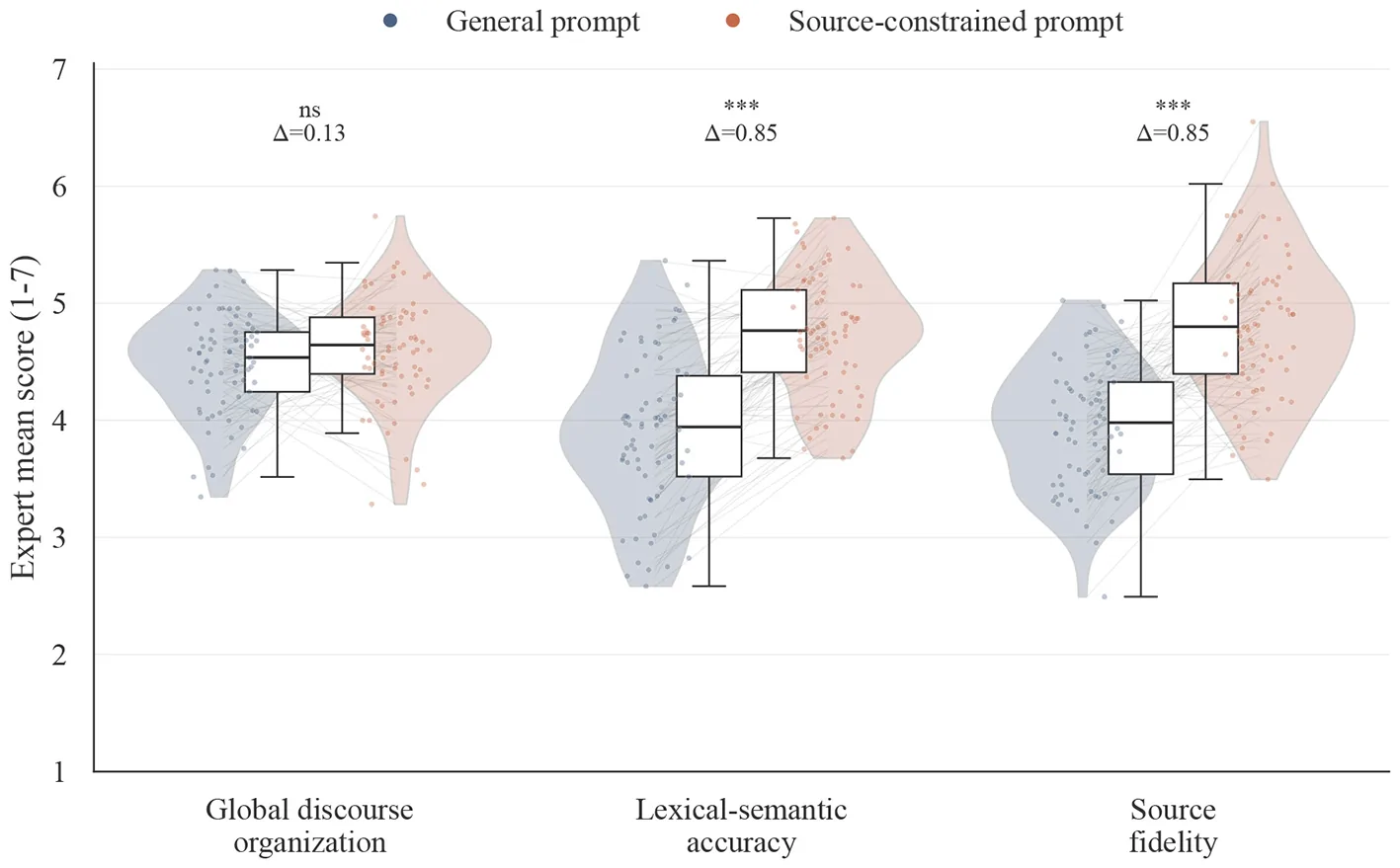
Distributions of global discourse organization, lexical-semantic accuracy, and source fidelity by generation condition (*n* = 73 participants per condition). Violin shapes show distributions, embedded box plots show medians and interquartile ranges, faint lines connect each participant's paired records, and dots represent individual AI-generated texts. Significance markers summarize paired tests: ns, *p* ≥ 0.05; *p < 0.05; **p < 0.01; ***p < 0.001.

### 5.2 Condition differences in AI-generated English text

The 0.131-point difference in global discourse organization was small, with a 95% CI of [-0.025, 0.287] and a small, non-significant difference (*t* = 1.673, *p* = 0.099, Cohen's *d*_*z*_ = 0.196, bootstrap 95% CI [-0.039, 0.422]). By contrast, lexical-semantic accuracy differed by 0.846 points (95% CI [0.688, 1.005]; *t* = 10.634, *p* < 0.001, Wilcoxon *p* < 0.001, Cohen's *d*_*z*_ = 1.245, bootstrap 95% CI [1.018, 1.565]) and source fidelity by 0.855 points (95% CI [0.662, 1.048]; *t* = 8.819, *p* < 0.001, Wilcoxon *p* < 0.001, Cohen's *d*_*z*_ = 1.032, bootstrap 95% CI [0.774, 1.353]). Overall quality differed by 0.613 points (*t* = 11.350, *p* < 0.001, Cohen's *d*_*z*_ = 1.328). The approximately 0.85-point differences in meaning-sensitive measures were therefore substantially larger than the 0.13-point difference in global organization, concentrating the condition contrast in lexical reference, concept boundaries, and source-based meaning (Figure 4).

**Figure 4:**
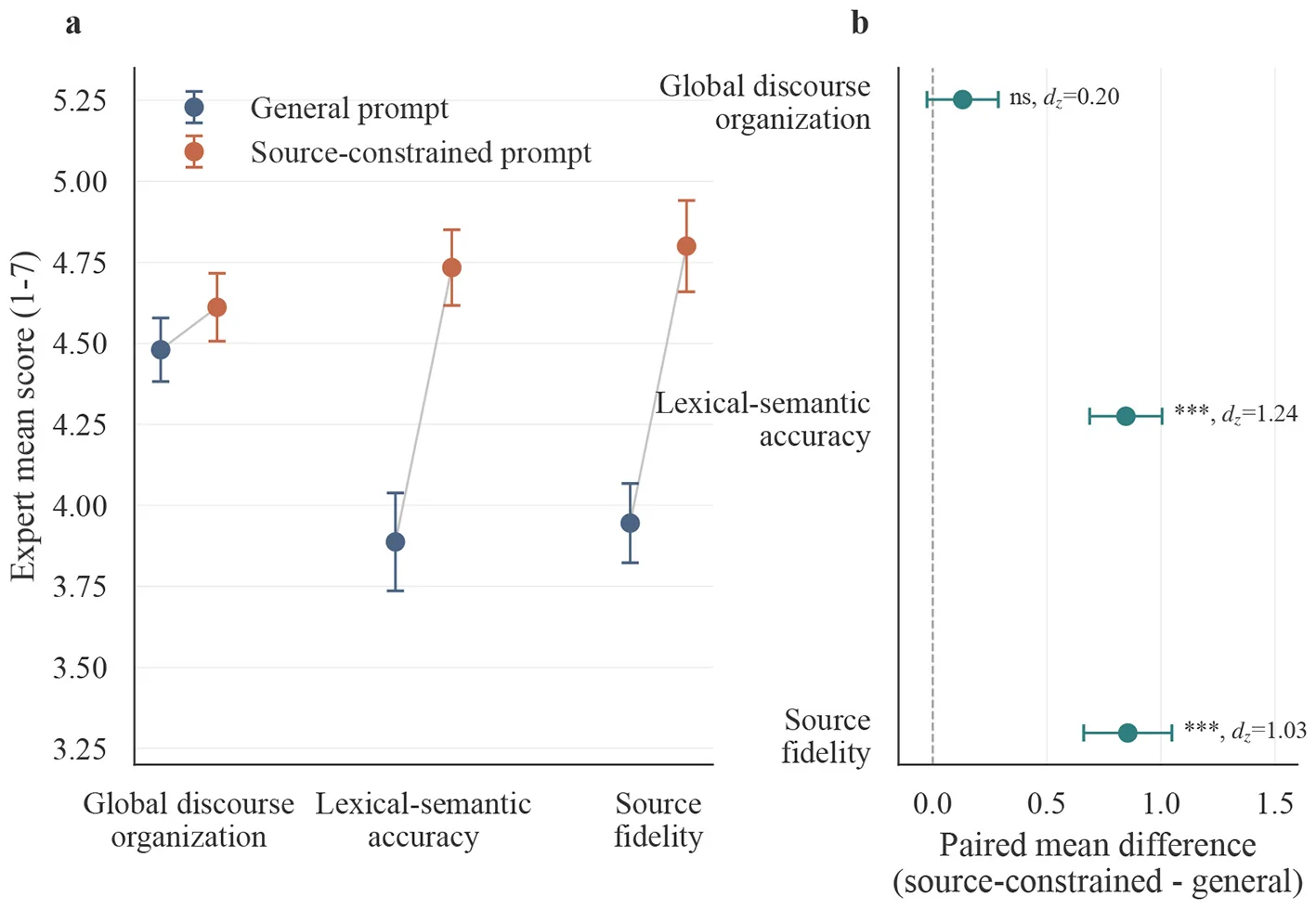
Condition means and paired differences in AI-generated English text. **(a)** Shows general and source-constrained means with 95% confidence intervals. **(b)** Shows within-participant differences calculated as source-constrained minus general scores, with 95% confidence intervals; Cohen's *d*_*z*_ is shown as a standardized paired effect size. Significance markers: ns, *p* ≥ 0.05; *p < 0.05; **p < 0.01; ***p < 0.001.

The same asymmetry appeared across writing sections and material packages (Table 6; Supplementary Table S24). Condition-by-section interactions were small for global discourse organization, *F*(2, 72) = 0.082, *p* = 0.921, lexical-semantic accuracy, *F*(2, 72) = 1.199, *p* = 0.307, and source fidelity, *F*(2, 72) = 1.077, *p* = 0.346. Section-specific differences were 0.162, 0.139, and 0.092 for global organization across Introduction, Methods, and Results and Discussion, respectively; 1.036, 0.773, and 0.734 for lexical-semantic accuracy; and 0.813, 1.040, and 0.704 for source fidelity. Additive estimates were *b* = 0.131 (95% CI [-0.028, 0.290]) for global organization, *b* = 0.846 (95% CI [0.685, 1.008]) for lexical-semantic accuracy, and *b* = 0.855 (95% CI [0.659, 1.051]) for source fidelity. The section-specific estimates and confidence intervals show that source-sensitive meaning preservation separated more strongly than paragraph-level organization across all three writing functions (Figure 4).

**Table 6:** Cluster-robust condition models for the three expert-rated language outcomes.

Outcome and term	Model component	*b*	SE	95% CI	*p*
Global discourse organization	Prompt, Introduction	0.162	0.127	[−0.086, 0.411]	0.200
	Prompt, Methods	0.139	0.168	[−0.191, 0.468]	0.408
	Prompt, Results and Discussion	0.092	0.121	[−0.145, 0.330]	0.447
	Additive prompt estimate	0.131	0.081	[−0.028, 0.290]	0.105
Lexical-semantic accuracy	Prompt, Introduction	1.036	0.158	[0.725, 1.346]	< 0.001
	Prompt, Methods	0.773	0.129	[0.520, 1.027]	< 0.001
	Prompt, Results and Discussion	0.734	0.133	[0.473, 0.994]	< 0.001
	Additive prompt estimate	0.846	0.082	[0.685, 1.008]	< 0.001
Source fidelity	Prompt, Introduction	0.813	0.178	[0.465, 1.161]	< 0.001
	Prompt, Methods	1.040	0.150	[0.746, 1.333]	< 0.001
	Prompt, Results and Discussion	0.704	0.188	[0.335, 1.072]	< 0.001
	Additive prompt estimate	0.855	0.100	[0.659, 1.051]	< 0.001

*n* = 146 text records from 73 participants. The interaction model included prompt condition × writing section and material-package fixed effects; residual *df* = 134 for the interaction models and *df* = 136 for the additive models. The interaction-model fits were *R*^2^ = 0.037 (adjusted *R*^2^ = −0.042) for global discourse organization, *R*^2^ = 0.442 (adjusted *R*^2^ = 0.396) for lexical-semantic accuracy, and *R*^2^ = 0.449 (adjusted *R*^2^ = 0.404) for source fidelity. The additive model omitted the unsupported interaction. Prompt condition was coded 0 = general prompt and 1 = source-constrained prompt. Introduction was the reference section and M1 the reference material package. Standard errors were clustered by participant. *b* denotes an unstandardized score-scale coefficient.

### 5.3 Lexical-semantic accuracy, source fidelity, and discourse organization

Lexical-semantic accuracy and source fidelity were closely related. Their bivariate correlation was *r* = 0.543, *p* < 0.001, and the association remained after controlling for word count, writing section, and material package (*b* = 0.522, SE = 0.070, 95% CI [0.385, 0.659], *p* < 0.001). Texts that preserved terms more accurately also tended to preserve variable identities, units, research objects, and source-supported claims.

Global discourse organization varied more independently. Its adjusted association with lexical-semantic accuracy was near zero (*b* = −0.008, SE = 0.065, 95% CI [-0.135, 0.120], *p* = 0.908), and its association with source fidelity was also small (*b* = 0.067, SE = 0.059, 95% CI [-0.049, 0.183], *p* = 0.258). The product of the two adjusted coefficients was 0.037, with a 5,000-resample participant-level bootstrap 95% CI of [-0.026, 0.106]. This coefficient product summarizes the observed cross-sectional associations and is reported as an exploratory coefficient product. Lexical-semantic accuracy was thus more closely related to source fidelity than to paragraph organization (Figure 5).

**Figure 5:**
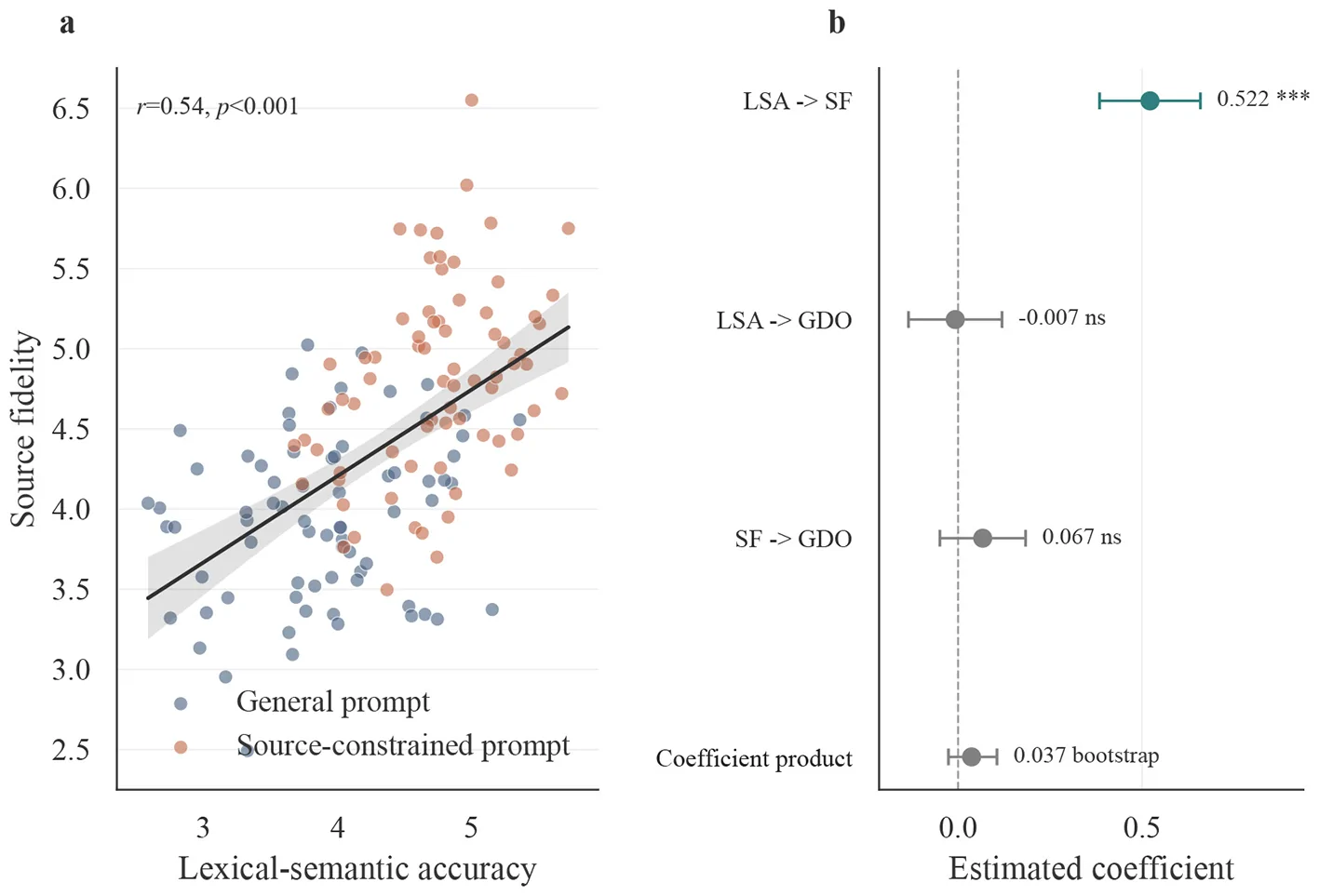
Lexical-semantic accuracy, source fidelity, and global discourse organization (*n* = 146 text records from 73 participants). **(a)** Shows the text-level lexical-semantic–source-fidelity association with an ordinary least squares fitted line and 95% confidence band. **(b)** Shows participant-clustered adjusted association coefficients and the coefficient product estimated from 5,000 participant-level bootstrap resamples. LSA, lexical-semantic accuracy; SF, source fidelity; GDO, global discourse organization. Significance markers: ns, *p* ≥ 0.05; *p < 0.05; **p < 0.01; ***p < 0.001.

### 5.4 Global judgment, localized problem marking, and written revision

Revision generally improved expert-rated quality. Across 146 judgments, participants' mean overall score was 4.55 and the expert mean was 4.41. Signed judgment bias averaged 0.138 (SD = 0.353): 98 scores exceeded the corresponding expert mean and 48 were lower. Global judgment discrepancy from Equation 1 averaged 0.284 and ranged from 0.007 to 1.468. Among the 73 selected revision records, mean overall quality increased from 4.57 (SD = 0.39) for the selected source text to 4.82 (SD = 0.46) for the revised text. Revision gain from Equation 2 averaged 0.256 (SD = 0.243, 95% CI [0.200, 0.313]); 63 participants improved and 10 declined, with no unchanged scores. Participants who selected a source-constrained text had source and revised means of 4.685 and 4.959 (gain *M* = 0.273), whereas the 15 participants who selected a general-prompt text had corresponding means of 4.102 and 4.293 (gain *M* = 0.191). The gain distribution had a median of approximately 0.25 (IQR approximately 0.31), ranged from approximately −0.35 to 0.85, and showed little skew. Selected-source and revised-text quality had no exact floor or ceiling observations; near-ceiling shares did not exceed 1.4% and 2.7%, respectively. In bivariate analyses, judgment discrepancy was negatively associated with gain (*r* = −0.240, *p* = 0.040; Figure 6a), while localized problem count was positively associated (*r* = 0.432, *p* < 0.001) (Supplementary Tables S25–S27).

**Figure 6:**
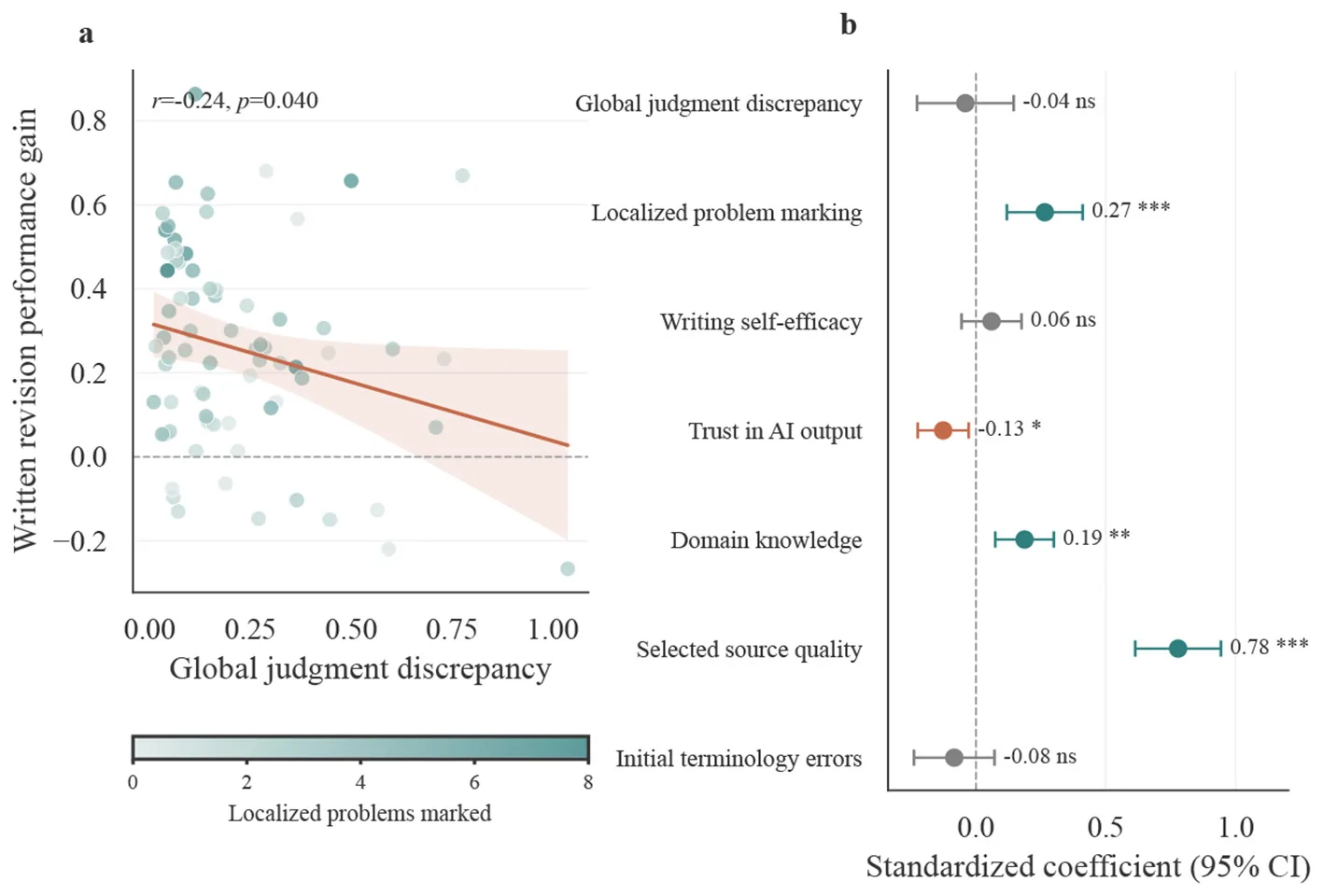
Global judgment discrepancy, localized problem marking, and written revision (*n* = 73). **(a)** Shows the bivariate association between selected-text judgment discrepancy and revision-performance gain; color represents localized problem count. **(b)** Shows standardized coefficients with HC3 robust 95% confidence intervals from the baseline-adjusted revised-quality model. Covariates were selected source quality, selected prompt condition, initial terminology-error count, writing section, and material package. ns, *p* ≥ 0.05; *p < 0.05; **p < 0.01; ***p < 0.001.

The 511 annotations were distributed across lexical, semantic, evidential, methodological, and discourse problems. Terminology issues were most frequent (*n* = 128, 25.0%), followed by disciplinary-fit issues (*n* = 81, 15.9%), overgeneralized claims (*n* = 76, 14.9%), discourse-coherence issues (*n* = 71, 13.9%), method/detail issues (*n* = 57, 11.2%), unsupported information (*n* = 51, 10.0%), and evidence-boundary issues (*n* = 47, 9.2%). The coding definitions and counting rules are provided in Supplementary Tables S16–S17.

The baseline-adjusted model of raw revised-text quality included 17 parameters for 73 participants (*R*^2^ = 0.861, adjusted *R*^2^ = 0.822; Table 7; Supplementary Table S23). Ramsey RESET yielded *F*(3, 53) = 1.08, *p* = 0.365, the joint quadratic-term test yielded *F*(5, 51) = 0.94, *p* = 0.463, and the Breusch–Pagan test yielded χ^2^(16) = 18.21, *p* = 0.312. Influence summaries identified 4–6 observations above the 4/*N* Cook's *D* threshold across the examined specifications, with maximum Cook's *D* = 0.18, maximum absolute studentized residual = 2.7, and maximum leverage = 0.43 (Supplementary Table S22). Localized problem count was positively associated with revised quality (*b* = 0.072, HC3 SE = 0.020, 95% CI [0.032, 0.111], *p* < 0.001), as was domain knowledge (*b* = 0.008, HC3 SE = 0.003, 95% CI [0.003, 0.013], *p* = 0.001). Trust in AI-generated language output was negatively associated with revised quality (*b* = −0.093, HC3 SE = 0.037, 95% CI [-0.165, −0.021], *p* = 0.012), while selected-source quality showed the largest positive association (*b* = 0.932, 95% CI [0.734, 1.129], *p* < 0.001). Global judgment discrepancy had no unique association after the other predictors entered the model (*b* = −0.090, 95% CI [-0.503, 0.322], *p* = 0.667). Standardized coefficients in Figure 6b show the same distinction: localized problem marking (β = 0.27, *p* < 0.001), domain knowledge (β = 0.19, *p* = 0.001), and trust (β = −0.13, *p* = 0.012) carried unique associations, whereas judgment discrepancy, self-efficacy, and initial terminology-error count did not.

**Table 7:** Participant-level models of written revision quality and revision-performance gain.

Term	*b*	HC3 SE	95% CI	*p*
Panel A. Raw revised-text overall quality, baseline adjusted				
Intercept	0.081	0.486	[−0.871, 1.032]	0.868
Selected source: general condition	−0.101	0.100	[−0.297, 0.094]	0.310
Writing section: Methods	−0.067	0.080	[−0.223, 0.089]	0.401
Writing section: Results and Discussion	−0.019	0.070	[−0.156, 0.118]	0.786
Material package M2	0.090	0.095	[−0.096, 0.276]	0.342
Material package M3	−0.016	0.133	[−0.276, 0.244]	0.903
Material package M4	−0.005	0.117	[−0.235, 0.224]	0.964
Material package M5	0.023	0.103	[−0.179, 0.224]	0.826
Material package M6	0.030	0.097	[−0.160, 0.220]	0.759
Material package M7	0.038	0.091	[−0.140, 0.216]	0.676
Global judgment discrepancy	−0.090	0.211	[−0.503, 0.322]	0.667
Localized problem count	0.072	0.020	[0.032, 0.111]	< 0.001
Writing self-efficacy	0.045	0.044	[−0.041, 0.130]	0.309
Trust in AI-generated language output	−0.093	0.037	[−0.165, −0.021]	0.012
Domain-knowledge score	0.008	0.003	[0.003, 0.013]	0.001
Selected source-text quality	0.932	0.101	[0.734, 1.129]	< 0.001
Initial terminology-error count	−0.030	0.029	[−0.087, 0.026]	0.291
Panel B. Algebraically equivalent revision-gain parameterization				
Intercept	0.081	0.486	[−0.871, 1.032]	0.868
Selected source: general condition	−0.101	0.100	[−0.297, 0.094]	0.310
Writing section: Methods	−0.067	0.080	[−0.223, 0.089]	0.401
Writing section: Results and Discussion	−0.019	0.070	[−0.156, 0.118]	0.786
Material package M2	0.090	0.095	[−0.096, 0.276]	0.342
Material package M3	−0.016	0.133	[−0.276, 0.244]	0.903
Material package M4	−0.005	0.117	[−0.235, 0.224]	0.964
Material package M5	0.023	0.103	[−0.179, 0.224]	0.826
Material package M6	0.030	0.097	[−0.160, 0.220]	0.759
Material package M7	0.038	0.091	[−0.140, 0.216]	0.676
Global judgment discrepancy	−0.090	0.211	[−0.503, 0.322]	0.667
Localized problem count	0.072	0.020	[0.032, 0.111]	< 0.001
Writing self-efficacy	0.045	0.044	[−0.041, 0.130]	0.309
Trust in AI-generated language output	−0.093	0.037	[−0.165, −0.021]	0.012
Domain-knowledge score	0.008	0.003	[0.003, 0.013]	0.001
Selected source-text quality	−0.068	0.101	[−0.266, 0.129]	0.499
Initial terminology-error count	−0.030	0.029	[−0.087, 0.026]	0.291

*n* = 73. Panel A: *R*^2^ = 0.861, adjusted *R*^2^ = 0.822, residual *df* = 56; Panel B: *R*^2^ = 0.497, adjusted *R*^2^ = 0.354, residual *df* = 56. Each model estimated 17 parameters. Reference categories were the source-constrained selected text, Introduction, and material package M1. Variance-inflation factors for non-intercept terms ranged from 1.10 to 2.52. Coefficients are unstandardized; HC3 heteroskedasticity-robust standard errors and confidence intervals are reported.

The gain-score specification had *R*^2^ = 0.497 and adjusted *R*^2^ = 0.354. Coefficients for localized problem marking (*b* = 0.072, 95% CI [0.032, 0.111], *p* < 0.001), domain knowledge (*b* = 0.008, 95% CI [0.003, 0.013], *p* = 0.001), trust (*b* = −0.093, 95% CI [-0.165, −0.021], *p* = 0.012), selected prompt condition (*p* = 0.310), and initial terminology-error count (*p* = 0.291) were identical to those in the baseline-adjusted model. The selected-source-quality coefficient was −0.068, compared with 0.932 in the baseline-adjusted model.

## 6 Discussion

The condition contrast was concentrated in lexical-semantic accuracy and source fidelity, whereas global discourse organization differed little between conditions. The source-constrained prompt contained explicit specifications of terms, objects, variables, units, result directions, and allowable claims. Texts generated in this condition showed larger differences from the general-prompt condition on lexical-semantic accuracy and source fidelity than on paragraph organization. Paragraph organization depends more on sequencing, coherence, and rhetorical progression, features that a general request for academic English may already elicit. AI-generated language can therefore be readable at the paragraph level while remaining vulnerable at the level of reference and evidence (Bender et al., 2021; Ji et al., 2023). The observed condition differences were larger for terminology and evidential scope than for rhetorical organization, extending disciplinary accounts of research writing (Hyland, 2008).

The close relation between lexical-semantic accuracy and source fidelity is consistent with the role of technical expressions in maintaining conceptual identity across Chinese source material and English research prose. Variables, treatments, species, indicators, and units constrain which entities a sentence denotes and which relations its claims can support. L2 lexical models explain why familiar English wording may still activate a meaning that is too broad or imprecise for the source concept (Kroll and Stewart, 1994; Jiang, 2000). Accurate lexical mappings may stabilize reference and contain interpretive scope, while source fidelity also depends on omissions, unsupported additions, causal commitment, and section function. The weaker associations involving paragraph organization indicate that proposition ordering and local coherence can remain effective even when a term or evidential boundary shifts. Source-based writing research likewise treats faithful paraphrase and rhetorical organization as related demands supported by different textual evidence (Keck, 2006; Wette, 2010).

Localized problem marking was more closely associated with subsequent revision than global judgment discrepancy. An overall score compresses impressions of language, content, and organization into a single response, whereas a marked terminology problem, overgeneralized claim, unsupported addition, evidence boundary, methodological detail, or coherence problem identifies a potential site of repair. This distinction connects explicit language knowledge with task demands (Ellis, 2004; Plonsky et al., 2020) and accords with accounts that distinguish evaluation from the localization of repairable material (Levelt, 1983; Kormos, 1999). Fluent AI text may encourage a broad fluency-based impression, allowing learners to judge a paragraph favorably without identifying the particular wording or claim that requires change. The association between localized problem count and revised quality is therefore consistent with a behavioral role for problem representation in revision, while the global discrepancy measure captures evaluative alignment at a different level.

Domain knowledge and trust in AI-generated language output were also associated with revised quality. Greater domain knowledge may provide richer semantic representations for checking whether variables, treatment relations, and claims remain faithful to the source. Trust showed the opposite association: higher reported trust accompanied lower-quality revision after the modeled covariates were held constant. Research on automation bias and algorithm appreciation offers relevant accounts of how people respond to automated suggestions (Parasuraman and Manzey, 2010; Logg et al., 2019), while AI-literacy frameworks place critical evaluation alongside knowledge of system capabilities and limitations (Long and Magerko, 2020; Ng et al., 2021). These findings position domain knowledge and trust as complementary resources in source-based revision: one supplies material for semantic comparison, while the other reflects a writer's stance toward generated language.

The fixed general-first, source-constrained-second sequence leaves condition differences partly intertwined with order and practice; counterbalanced designs would separate these sources of variation. Because participants selected the paragraph to revise, the two selector groups were unequal, and assigned-source designs would provide a more direct comparison of revision outcomes. Replication across AI platforms and beyond agricultural and forestry students would test the generality of the pattern, while independent proficiency measures and process tracing could show how writers inspect terms, compare claims with sources, and transform marked problems into revisions. These directions reinforce the central role of source comparison, localized problem detection, and precise lexical-semantic monitoring in the revision of AI-generated English.

## 7 Conclusion

Texts generated under the source-constrained condition showed higher meaning-sensitive scores than texts generated under the general condition: lexical-semantic accuracy and source fidelity differed by approximately 0.85 points, while global organization differed by approximately 0.13 points. More accurate terminology accompanied closer preservation of variables, units, research objects, and evidential scope. During revision, localized problem marking carried more information about subsequent written quality than agreement with an expert's global score, and domain knowledge and trust in AI-generated language output added further individual differences. Source comparison, localized problem detection, and precise lexical-semantic monitoring thus provide practical foundations for revising fluent AI-generated English with greater fidelity to disciplinary meaning.

## Data Availability

The raw data supporting the conclusions of this article will be made available by the authors, without undue reservation.
