# Supplementary material for: Monitoring AI-generated English research texts: lexical-semantic accuracy, source fidelity, and revision performance
Source: Front Psychol. 2026 Oct 19;17:1953783. doi: 10.3389/fpsyg.2026.1953783 (PMC13724414; doi:10.3389/fpsyg.2026.1953783)
Supplement: Supplementary file 1 [file Supplementary_file_1.pdf]

frontiers

Supporting Information

1 SUPPORTING TABLES

Table S1: Measurement tools and overall structure.

Tool or variable	Design and recorded indicator
Writing self-efficacy	Four-item, five-point Likert scale; the mean of the four recorded item scores was used as the total score.
Trust in AI-generated language output	Four-item, five-point Likert scale; TR3 was reverse scored before the four-item mean was calculated.
AI-use frequency	One five-level frequency item.
Domain knowledge	Twenty single-choice questions; five points per item and a total score from 0 to 100.
Participant overall-quality judgment	One 1–7 rating for each AI-generated text.
Global discourse organization	Independent 1–7 ratings by three experts.
Lexical-semantic accuracy	Independent 1–7 ratings by three experts.
Source fidelity	Independent 1–7 ratings by three experts.
Overall written quality	Independent 1–7 ratings by three experts.
Terminology-error coding	Two coders independently coded each term unit using seven error categories.
Localized problem marking	Participants marked problems directly in the text; the markings were subsequently classified into seven problem categories.

Table S2: Writing self-efficacy scale items.

Item	Item wording	Score
WSE1	I can accurately express the main concepts and variables in my field of research in English.	1–5
WSE2	When Chinese research materials contain multiple experimental conditions, I can clearly express their relationships in English writing.	1–5
WSE3	I can organize an English research paragraph with clear structure and logical coherence.	1–5
WSE4	When an English expression differs from the original Chinese material, I can usually identify and revise it.	1–5

Instructions: Participants rated each statement according to their actual experience completing English research-writing tasks in agriculture, forestry, and related fields. Response anchors were 1 = strongly disagree, 2 = disagree, 3 = uncertain, 4 = agree, and 5 = strongly agree.

Table S3: Writing self-efficacy scoring rules.

Item	Reference rule
Number of items	4
Reverse-scored items	None
Item range	1–5
Total score	Mean of the four items
Theoretical range	1.00–5.00
Missing-data rule	If no more than one item is missing, use the mean of the available items; if two or more items are missing, the scale score is missing.
Meaning of a high score	Higher self-efficacy for completing source-constrained English research writing.

Table S4: Trust in AI-generated language output scale items.

Item	Item wording	Scoring
TR1	When AI-generated English expressions read fluently, I usually regard most of the information as reliable.	1–5
TR2	I generally believe that AI can accurately convert Chinese research concepts into English expressions.	1–5
TR3	For experimental conditions, numerical values, units, and relationships among variables, I believe it is still necessary to check the AI text item by item against the original material.	1–5, reverse scored
TR4	If an AI-generated sentence follows academic English conventions, I am usually willing to retain its original wording.	1–5

Instructions: The statements concerned participants' usual views of English research text generated by generative AI. Participants rated each statement according to their actual experience.

Table S5: Trust in AI-generated language output scoring rules.

Item	Reference rule
Scale and item range	Four items rated on a five-point Likert scale (1–5)
Positive items	TR1, TR2, and TR4
Reverse-scored item	TR3; 1 becomes 5, 2 becomes 4, 3 remains 3, 4 becomes 2, and 5 becomes 1
Total score and direction	Mean of the four item scores after reverse scoring; higher scores indicate greater general trust in AI-generated research language.

Table S6: AI-use frequency item.

Item or score	Content
Question	During the past six months, how often have you used generative AI to support English learning, English writing, or text revision?
1	Never
2	Less than once a month
3	1–3 times per month
4	1–3 times per week
5	Four or more times per week
Scoring	Directly use the 1–5 score.

Table S7: Domain knowledge test items and answer key.

Item	Question	Options (A–D)	Answer
DK1	Under salt stress, MDA is usually used as an indicator of which type?	A chlorophyll synthesis; B membrane lipid peroxidation; C nitrogen uptake; D photoperiod	B
DK2	SOD, POD, and CAT in plant-stress research usually belong to which category?	A antioxidant defense; B nutrient uptake; C reproductive development; D morphogenesis	A

Table S7: Domain knowledge test items and answer key (continued).

Item	Question	Options (A–D)	Answer
DK3	After nitrogen application increases, crop yield no longer increases significantly. The most appropriate explanation is:	A yield is necessarily negatively related to nitrogen fertilizer; B the marginal yield response may weaken; C nitrogen fertilizer is completely ineffective; D all treatments are exactly the same	B
DK4	Nitrogen-use efficiency mainly describes:	A the utilization effect produced per unit of nitrogen input; B total soil nitrogen concentration; C plant water content; D photosynthetic rate	A
DK5	An increase in soil pH from 4.8 to 5.6 indicates:	A greater acidity; B lower acidity; C necessarily lower soil salinity; D necessarily greater organic matter	B
DK6	In a biochar-amendment study, the main purpose of a 0% addition group is to:	A increase sample size; B provide a control baseline; C reduce error; D increase significance	B
DK7	Net photosynthetic rate most directly reflects:	A net carbon assimilation; B root respiration; C soil water content; D nitrogen-fertilizer concentration	A
DK8	Stomatal conductance is directly related mainly to:	A gas exchange through stomata; B root elongation; C seed germination; D soil mineralization	A
DK9	In a plant-pathogen infection experiment, the main function of an uninoculated treatment is to:	A provide an experimental control; B increase pathogen concentration; C change the species; D produce random error	A

Table S7: Domain knowledge test items and answer key (continued).

Item	Question	Options (A–D)	Answer
DK10	In plant-stress research, ROS are:	A reactive oxygen species; B root organic matter; C relative permeability; D a soil oxidation layer	A
DK11	Relative water content is usually used to describe plant:	A tissue water status; B soil nitrogen content; C leaf age; D pathogen number	A
DK12	Increased electrolyte leakage usually indicates that:	A cell-membrane integrity has been affected; B photosynthesis has necessarily increased; C root length has increased; D soil pH has increased	A
DK13	The Shannon diversity index is mainly used to represent:	A community diversity; B soil density; C plant height; D leaf area	A
DK14	If plant diversity is significantly positively correlated with microbial diversity, the direct conclusion is:	A the two are statistically associated; B plant diversity necessarily increases microbial diversity; C microbes necessarily increase plant diversity; D a biological mechanism has been proven	A
DK15	When an observational study finds that X and Y are correlated, the most cautious scientific wording is:	A X causes Y; B X is associated with Y; C X determines Y; D Y is produced by X	B
DK16	An experiment has four treatments with six independent replicates per treatment. The total number of experimental units is:	A 10; B 20; C 24; D 30	C
DK17	In a research paper, translating significantly correlated as caused mainly creates which problem?	A a tense error; B an expanded causal commitment; C a spelling error; D a unit error	B

Table S7: Domain knowledge test items and answer key (continued).

Item	Question	Options (A–D)	Answer
DK18	The source states 100 mmol L^{-1} NaCl, but the English text says 100 mmol NaCl. What is mainly lost?	A species information; B concentration-unit information; C treatment direction; D time information	B
DK19	The source limits the research object to maize seedlings, but the AI text changes it to plants. This is mainly:	A expansion of the conceptual range; B a numerical error; C a grammatical error; D a tense error	A
DK20	A Results paragraph reports only that an indicator increased, but adds because gene X was activated when the source contains no such evidence. This is:	A a reasonable synonym; B an addition of unsupported information; C a formatting change; D unit standardization	B

Table S8: Domain knowledge scoring rules.

Item	Reference setting
Number of items	20
Format	Single-choice questions with four options
Correct answer	5 points
Incorrect or unanswered	0 points
Total score	0–100
Expected completion time	12–15 min
Order	Completed before AI-text generation and evaluation tasks
Reference use	Not permitted
AI use	Not permitted
Feedback	Correct answers were not provided before the main experiment ended
Expected difficulty	Moderate; target mean accuracy approximately 0.65–0.75
Reference item analysis	Item difficulty approximately 0.42–0.88; item-total correlation preferably ≥ 0.20
Scoring variable	Number correct $\times$ 5

Table S9: Participant global-quality judgment anchors.

Score	Anchor
1	Very poor: the paragraph contains many obvious problems and is difficult to use as research English.
2	Poor: the paragraph contains multiple serious language, terminology, or content problems.
3	Relatively low: the paragraph is basically understandable but has clear accuracy or organization problems.
4	Moderate: the paragraph is broadly acceptable but still contains several problems requiring revision.
5	Relatively good: the paragraph is generally accurate and clear, with limited problems.
6	Very good: language and content are reliable, with only a few minor revisions needed.
7	Excellent: language, meaning, and disciplinary expression are highly appropriate, with almost no revision needed.

Participants gave one overall-quality score for each AI-generated text.

Table S10: Global discourse organization scoring anchors.

Score	Anchor
1	The paragraph lacks a clear topic and organization; sentence relations are confused; information order seriously obstructs understanding.
2	Organization has serious problems; some local content is understandable, but cohesion and information hierarchy are weak.
3	A basic paragraph structure is present, but topic development, cohesion, or information arrangement has clear deficiencies.
4	Overall structure is broadly reasonable and the main information is identifiable, but some sentence links or levels of information need improvement.
5	Organization is clear; sentence relations are generally coherent; the rhetorical function of the paper section is represented reasonably well.
6	Structure is clear and coherent; information hierarchy is appropriate; academic style and the target section are highly compatible.
7	Paragraph organization is highly mature; topic, development, cohesion, information hierarchy, and academic style are all excellent.

Table S11: Lexical-semantic accuracy scoring anchors.

Score	Anchor
1	Many core terms, variables, units, or concept expressions are incorrect, seriously changing the original meaning.
2	Multiple key concepts contain clear mistranslations or concept drift.
3	Some core terms are correct, but several semantic problems affect interpretation of meaning.
4	Main concepts are broadly preserved, but a small number of terminology or concept-boundary problems remain.
5	Most terms and variables are accurate, with only minor or local semantic deviations.
6	Technical terms, variables, units, and conceptual ranges are almost completely accurate.
7	All important terms, variables, species, indicators, units, and concept boundaries are accurately retained.

Table S12: Source fidelity scoring anchors.

Score	Anchor
1	Much of the content is inconsistent with the source; the research object, results, or conclusion is clearly changed.
2	Multiple serious omissions, additions, or errors in evidential scope are present.
3	Some core information is retained, but important conditions, result directions, or conclusion range are clearly changed.
4	Main information is broadly retained, with a small number of omissions, additions, or expanded inferences.
5	Most research objects, conditions, and results are accurately retained, with only minor changes in scope.
6	Research objects, experimental conditions, result direction, evidential boundaries, and section function are highly faithful.
7	The text is highly consistent with the source, with no substantive omissions, unsupported additions, causal strengthening, or expansion of evidential scope.

Table S13: Overall written quality scoring anchors.

Score	Anchor
1	The paragraph cannot be used as academic text and requires extensive rewriting.
2	Multiple serious problems are present and extensive revision is required.

Table S13: Overall written quality scoring anchors (continued).

Score	Anchor
3	The paragraph is basically understandable, but accuracy and academic expression are insufficient.
4	The paragraph reaches a basically acceptable level but still requires substantial revision.
5	Overall quality is good; the main content is accurate and only limited revision is needed.
6	The paragraph is high-quality academic writing with only minor problems.
7	Language, meaning, organization, and disciplinary fit are all excellent, and the paragraph essentially needs no revision.

Table S14: Terminology-error coding manual.

Code	Category	Operational definition	Inclusion standard	Exclusion	Example
TE1	Mistranslation	English corresponds to an incorrect source concept.	Source concept A is explicitly translated as concept B.	Do not include an expression that is merely unnatural when the meaning is correct.	”root vitality” when ”root activity” is required.
TE2	Vague generalization	A broader superordinate concept replaces a more specific term.	”maize seedlings” becomes ”plants”; ”MDA” becomes ”oxidative indicators.”	Do not include a legitimate abbreviation when reference is unambiguous.	”wheat” becomes ”crops.”
TE3	Collocation problem	A single word has an approximately relevant meaning, but its combination does not follow professional English convention and affects disciplinary interpretation.	An inappropriate technical noun–verb or noun–modifier combination.	Do not include a general stylistic problem.	”perform photosynthesis rate.”

Table S14: Terminology-error coding manual (continued).

Code	Category	Operational definition	Inclusion standard	Exclusion	Example
TE4	Unit/indicator error	A numerical value, unit, concentration form, or indicator identity changes.	Concentration unit is omitted or the indicator identity changes.	Do not include a format-only change with exactly equivalent numerical meaning.	”100 mmol L^{-1}” becomes ”100 mmol.”
TE5	Species-expression error	The identity of a species, crop, rootstock, or biological category is expressed incorrectly.	”maize” becomes ”wheat”; ”rootstock” becomes ”cultivar.”	Do not include equivalent scientific-name and common-name expressions.	”maize seedlings” becomes ”rice seedlings.”
TE6	Concept drift	Surface wording remains related, but the conceptual boundary or relation moves substantially.	A correlation is expressed as an effect, or water status as water-use efficiency.	Do not include a legitimate paraphrase that preserves the conceptual boundary.	”relative water content” becomes ”water-use efficiency.”
TE7	Unsupported information	The text adds a terminological fact, mechanism, condition, or relationship absent from the source.	A new gene, enzyme, mechanism, or treatment condition is added.	Do not include a connective or paraphrase that adds no fact.	Adding ”through ABA signaling” without mechanism data.

Table S15: Terminology coding rules.

Rule	Specification
Coding unit	One pre-identified term, variable, unit, species name, or concept expression constitutes one term unit.
Unit without an error	Record “0 = no terminology error.”
Unit with an error	Record one primary error code.
Two possible categories	Prioritize the category that most directly changes meaning.
Mistranslation with generalization	Prioritize Mistranslation; use Vague generalization when the range becomes broader but the basic category remains unchanged.
Correct number with omitted unit	Unit/indicator error.

Table S15: Terminology coding rules (continued).

Rule	Specification
Species error	Prioritize Species-expression error.
Mechanism absent from source	Unsupported information.
Repeated problem across sentences	Code each independent occurrence as a separate term unit.
Spelling error	If terminology identification is unaffected, do not count a terminology error; if term identity changes, code the relevant semantic category.

Table S16: Participant-marked problem categories.

Code	Category	Typical identification basis
PM1	Terminology issue	The participant points out a problem in a technical term, variable name, species, or unit expression. Example: “This term is translated inaccurately.”
PM2	Disciplinary-fit issue	The expression is grammatical but does not fit disciplinary convention, research object, or section function. Example: a Methods paragraph written as a mechanism-focused Discussion paragraph.
PM3	Overgeneralized claim	The AI text expands a local or conditional conclusion in the source into a general conclusion. Example: “under this treatment” becomes “in plants generally.”
PM4	Discourse-coherence issue	The participant identifies a problem in sentence logic, paragraph structure, or information order. Example: results appear in a sequence that does not match the topic sentence.
PM5	Method/detail issue	A method, treatment condition, time, replicate number, or experimental detail is incorrect. Example: 7 d becomes 14 d.
PM6	Unsupported information	The AI text adds a fact, explanation, or mechanism absent from the source. Example: a new gene, signaling pathway, or biological explanation.
PM7	Evidence-boundary issue	The text changes evidential strength or inferential range. Example: “was associated with” becomes “caused” or “may” becomes “demonstrates.”

Table S17: Localized problem-count calculation rules.

**Situation**	**Counting rule**
One independent marked fragment with an identified problem	Count 1.
One mark spanning a continuous phrase or sentence and referring to one problem	Count 1.
One fragment with two explicitly different problems	May count 2.
Underlining only, with no interpretable problem direction	The coder judges from the marking location; if the direction cannot be determined, do not count it.
The same error marked twice	Count the same location once.
The same error appearing at different locations	Count each independent location separately.
Total variable	The sum of valid markings made by each participant in the text evaluated by that participant.
Classification variable	Retain the PM1–PM7 category for each marking.

Table S18: Terminology coder training procedure.

**Stage**	**Procedure**
Number of coders	2
Initial manual	The manual provided TE1–TE7 definitions, decision rules, and examples.
First training round	The coders jointly studied approximately 20 example term units.
Independent practice	Each coder independently coded 60 additional practice units.
Difference discussion	The coders discussed the basis for each disagreement item by item.
Manual revision	Priorities were fixed for species errors, concept drift, generalization, and unsupported information.
Second practice round	Each coder independently coded another 40 units.
Entry standard for formal coding	Krippendorff's α reached approximately .75 or above.
Formal coding	All 562 formal term units were independently coded by both coders.
Agreement statistic	Krippendorff's α.
Current manuscript result	$\alpha = .803$.
Disagreement resolution	After agreement statistics were completed, both coders rechecked disagreements against the source material; unresolved cases were adjudicated by a third disciplinary expert.

Table S19: Coding decision rules for ambiguous cases.

Ambiguous case	Final coding decision
“maize seedlings” to “maize plants”	Vague generalization when the research-stage identity is important.
“maize seedlings” to “plants”	Vague generalization with greater severity.
“rice root growth” to “rice growth”	Vague generalization.
“soil microbial community” to “soil microorganisms”	Use the contextual decision; if community-level meaning is lost, code Concept drift.
“was associated with” to “increased”	Evidence-boundary issue when the wording implies directional action.
“was associated with” to “caused”	Evidence-boundary issue.
Correct numerical value with an equivalent international unit format	Do not count an error.
mmol L^{-1} to mM with an equivalent value	Do not count an error.
kg N ha^{-1} to kg ha^{-1}	Unit/indicator error.
No mechanism in the Chinese source, mechanism added in English	Unsupported information.
One minor background detail omitted by AI	Usually not a terminology error; it may affect the Source Fidelity score.
One key treatment condition omitted by AI	Method/detail issue; it also lowers the Source Fidelity score.
Spelling error without a change in meaning	Do not count a terminology error; it may affect Overall Quality.
Spelling error that forms another technical term	Mistranslation or Concept drift, as appropriate.

Table S20: Complete general and source-constrained prompt templates.

Condition	Complete prompt template
General prompt	Please write an approximately 170–210-word English research-paper paragraph based on the Chinese research material provided below, and make it suitable for the specified paper section. Use formal, clear, coherent academic English with reasonable links between sentences and an academic register. Output only the English paragraph. Do not add a heading, bullets, explanation, references, or any other additional content. Target paper section: [Introduction / Methods / Results and Discussion]. Chinese source material: [insert the full text of the corresponding M1–M7 material].

Table S20: Complete general and source-constrained prompt templates (continued).

Condition	Complete prompt template
Source-constrained prompt	Please write an approximately 170–210-word English research-paper paragraph strictly based on the Chinese research material provided below, and make it suitable for the specified paper section. The paragraph must: accurately retain the research object and its species or material identity; retain all experimental variables and indicators; retain treatment groups, experimental conditions, and comparison relations; keep all numerical values and units consistent with the source; preserve the direction of results and between-group relations; prioritize the supplied technical terms; add no experimental results, mechanisms, causes, or background facts absent from the source; preserve correlational wording; keep conclusions within the source-supported range; and match the rhetorical function of the specified paper section. Use formal, coherent academic English. Output only the English paragraph. Do not add a heading, bullets, explanation, references, or other additional content. Target paper section: [Introduction / Methods / Results and Discussion]. Required technical terms: [corresponding terminology list]. Chinese source material: [insert the full text of the corresponding M1–M7 material].

Table S21: AI generation environment and session-control records.

Item	Recorded setting
AI platform	Doubao web version, standard text-dialogue interface.
Doubao version	The web interface did not expose a stable backend model ID; no model-version label is assigned.
Generation dates	23–27 March 2026, Beijing time (UTC+8).
Access method	Direct browser access to the web interface; no Volcengine API or other programmatic interface.
API endpoint	Not applicable.
API key	Not applicable.
Temperature	Not set or exposed by the web interface.
Top-p	Not set or exposed by the web interface.
Top-k	Not set or exposed by the web interface.
Maximum tokens	Not set manually; approximate length was controlled by the 170–210-word prompt instruction.
Random seed	Not available for setting.
Frequency penalty	Not set or exposed by the web interface.
Presence penalty	Not set or exposed by the web interface.
System prompt	No researcher-defined system prompt.
Web search	Not enabled; the task permitted use of the supplied Chinese material only.

Table S21: AI generation environment and session-control records (continued).

Item	Recorded setting
File retrieval or RAG	Not used.
Uploaded attachments	None; Chinese material was pasted directly into the prompt box.
Personalization memory	Not used.
Input language	Chinese instructions and Chinese source material.
Output language	English.
Output format	One academic English paragraph; no heading, bullets, explanation, or references.
Target length	Approximately 170–210 English words.
General-prompt condition	Source material, target paper section, and general academic-writing requirements were provided.
Source-constrained condition	Object, variables, treatments, units, result direction, technical terms, evidence boundary, and section-function constraints were added.
Condition order	General prompt followed by Source-constrained prompt.
Random order	No.
Conversation isolation	Each formal generation used a new independent conversation; the two conditions did not share previous model responses.
History length	Zero prior turns before each formal generation; the current prompt produced one model output.
Model follow-up	No follow-up question was sent to the model.
Content-based regeneration	0.
Technical retries	Reference record: 6 retries among 146 generation requests; 4 network interruptions and 2 truncated outputs.
Technical retry rule	The identical prompt was resubmitted without modification; only the first complete output was retained.
Regenerate button	Not used for content quality; technical failures were handled by resubmitting the same prompt.
Multiple-output selection	Prohibited.
Human editing before evaluation	AI output was not edited before the evaluation stage.
Original-record fields	Participant ID, material ID, condition, complete prompt, original output, timestamp, and retry flag.

Table S22: Regression-model diagnostics and robustness checks.

**Diagnostic**	**Result**
Ramsey RESET	$F(3, 53) = 1.08$, $p = .365$
Quadratic-term joint test	$F(5, 51) = 0.94$, $p = .463$
Breusch–Pagan test	$\chi^2(16) = 18.21$, $p = .312$
Variance-inflation factor	1.10–2.52
Maximum Cook's distance	0.18
Observations with Cook's $D > 4/N$	4–6 observations
Maximum absolute studentized residual	2.7
Maximum leverage	0.43

Table S23: Participant-level revision model.

**Predictor**	b	**HC3 SE**	**95% CI**	p
Intercept	0.081	0.486	[-0.871, 1.032]	.868
Selected source: general	-0.101	0.100	[-0.297, 0.094]	.310
Methods	-0.067	0.080	[-0.223, 0.089]	.401
Results and Discussion	-0.019	0.070	[-0.156, 0.118]	.786
Material M2	0.090	0.095	[-0.096, 0.276]	.342
Material M3	-0.016	0.133	[-0.276, 0.244]	.903
Material M4	-0.005	0.117	[-0.235, 0.224]	.964
Material M5	0.023	0.103	[-0.179, 0.224]	.826
Material M6	0.030	0.097	[-0.160, 0.220]	.759
Material M7	0.038	0.091	[-0.140, 0.216]	.676
Global judgment discrepancy	-0.090	0.211	[-0.503, 0.322]	.667
Localized problem count	0.072	0.020	[0.032, 0.111]	$<$ .001
Writing self-efficacy	0.045	0.044	[-0.041, 0.130]	.309
Trust in AI-generated language output	-0.093	0.037	[-0.165, -0.021]	.012
Domain knowledge	0.008	0.003	[0.003, 0.013]	.001
Selected source-text quality	0.932	0.101	[0.734, 1.129]	$<$ .001
Initial terminology-error count	-0.030	0.029	[-0.087, 0.026]	.291

Note. The model predicts raw revised-text overall quality for $N = 73$ participants. HC3 SE = heteroskedasticity-consistent standard error. The model also included the displayed writing-section and material-package indicators; the reference categories were Introduction and M1.

Table S24: Prompt effects across condition models.

**Outcome**	**Section / model**	b	**SE**	**95% CI**	p
Global discourse organization	Introduction	0.162	0.127	[-0.086, 0.411]	.200
	Methods	0.139	0.168	[-0.191, 0.468]	.408
	Results and Discussion	0.092	0.121	[-0.145, 0.330]	.447
	Additive estimate	0.131	0.081	[-0.028, 0.290]	.105
Lexical-semantic accuracy	Introduction	1.036	0.158	[0.725, 1.346]	$<$ .001

Table S24: Prompt effects across condition models (continued).

**Outcome**	**Section / model**	b	**SE**	**95% CI**	p
	Methods	0.773	0.129	[0.520, 1.027]	$< .001$
	Results and Discussion	0.734	0.133	[0.473, 0.994]	$< .001$
	Additive estimate	0.846	0.082	[0.685, 1.008]	$< .001$
Source fidelity	Introduction	0.813	0.178	[0.465, 1.161]	$< .001$
	Methods	1.040	0.150	[0.746, 1.333]	$< .001$
	Results and Discussion	0.704	0.188	[0.335, 1.072]	$< .001$
	Additive estimate	0.855	0.100	[0.659, 1.051]	$< .001$

Note. $n = 146$ text records from 73 participants. The interaction models included prompt condition $\times$ writing section and material-package fixed effects; the additive estimate omitted the interaction. Prompt condition was coded 0 = preceding general prompt and 1 = later source-constrained prompt. Standard errors were clustered by participant.

Table S25: Descriptive characteristics by selected source condition.

**Variable**	**Selected source-constrained** ($n = 58$)	**Selected general** ($n = 15$)
Selected source quality	4.685	4.102
Revised quality	4.959	4.293
Revision gain	0.273	0.191
Domain knowledge	$\approx$ 70.8 (10.3)	$\approx$ 67.2 (11.0)
Writing self-efficacy	$\approx$ 3.24 (0.61)	$\approx$ 3.14 (0.66)
Trust in AI-generated language output	$\approx$ 3.18 (0.62)	$\approx$ 3.41 (0.65)
Judgment discrepancy of selected text	$\approx$ 0.25 (0.21)	$\approx$ 0.36 (0.31)
Localized problem count	$\approx$ 2.4 (1.7)	$\approx$ 3.3 (1.9)
Initial terminology errors	$\approx$ 1.2 (1.3)	$\approx$ 2.3 (1.6)
AI-use frequency	$\approx$ 2.7 (0.9)	$\approx$ 2.6 (0.9)

Note. Values in parentheses are standard deviations. The approximation sign identifies values supplied as rounded descriptive summaries. No inferential comparison between the self-selected groups is implied.

Table S26: Distribution of revision-performance gain.

**Statistic**	**Value**
N	73
Mean	0.256
SD	0.243
95% CI of mean	[0.200, 0.313]
Gain > 0	63 (86.3%)
Gain $= 0$	0 (0.0%)
Gain < 0	10 (13.7%)
Source-constrained selectors, mean	0.273
General selectors, mean	0.191

Table S26: Distribution of revision-performance gain (continued).

Statistic	Value
Median	≈ 0.25
First quartile	≈ 0.10
Third quartile	≈ 0.41
Interquartile range	≈ 0.31
Minimum	≈ -0.35
Maximum	≈ 0.85
Skewness	≈ 0.05–0.25
Bootstrap 95% CI for mean gain	$\approx [0.20, 0.31]$

Table S27: Ceiling and floor assessment.

Outcome	Floor (=1)	Near floor (≤ 1.5)	Ceiling (=7)	Near ceiling (≥ 6.5)
Selected-source overall quality	0%	0%	0%	0–1.4%
Revised-text overall quality	0%	0%	0%	0–2.7%
Global discourse organization	0%	Very low	0%	Very low
Lexical-semantic accuracy	0%	Very low	0% or very few	Low
Source fidelity	0%	Very low	0% or very few	Low

Note. Qualitative descriptors and percentage ranges reproduce the available assessment and are not converted into exact counts.
